# In Vivo Imaging of Immune Rejection of MIN6 Cells Transplanted in C3H Mice

**DOI:** 10.3390/cells13121044

**Published:** 2024-06-17

**Authors:** Jyuhn-Huarng Juang, Chen-Ling Chen, Chen-Wei Kao, Shu-Ting Wu, Chia-Rui Shen

**Affiliations:** 1 Division of Endocrinology and Metabolism, Department of Internal Medicine, Center for Tissue Engineering, Chang Gung Memorial Hospital, Taoyuan 33305, Taiwan; jenny74513@gmail.com (C.-L.C.); lian8807111@gmail.com (C.-W.K.); 2 Department of Medical Biotechnology and Laboratory Science, College of Medicine, Chang Gung University, Taoyuan 33302, Taiwan; proteinwhite@livemail.tw; 3 Department of Ophthalmology, Chang Gung Memorial Hospital, Taoyuan 33305, Taiwan; 4 R&D Center of Biochemical Engineering Technology, Department of Chemical Engineering, Ming Chi University of Technology, New Taipei 24301, Taiwan

**Keywords:** MIN6 cells, transplantation, C3H mice, immune rejection, bioluminescence imaging

## Abstract

Recently, we successfully utilized noninvasive magnetic resonance and bioluminescence imaging to track MIN6 cells subcutaneously transplanted in immunocompromised nude mice for up to 64 days. In this study, we further used bioluminescence imaging to investigate the immune rejection of MIN6 cells in immunocompetent C3H mice. A total of 5 × 10^6^ luciferase-transfected MIN6 cells were implanted into the subcutaneous space of each nude or C3H mouse. After transplantation, hypoglycemia and persistent bioluminescence signals were observed in eight of eight (100%) nude mice and five of nine (56%) C3H mice (*p* < 0.05). We then presensitized a group of C3H mice with C57BL/6 spleen cells just prior to transplantation (n = 14). Interestingly, none of them had hypoglycemia or persistent bioluminescence signals (*p* < 0.01 vs. C3H mice without presensitization). Histological examination of the grafts revealed a lack or minimal presence of insulin-positive cells in recipients without hypoglycemia and persistent bioluminescence signals. In contrast, recipients with hypoglycemia and persistent bioluminescence signals showed a significant presence of insulin-positive cells in their grafts. Our results indicate that rejection of MIN6 cells occurred in C3H mice and could be enhanced by presensitization with C57BL/6 spleen cells and that bioluminescence imaging is a useful noninvasive tool for detecting rejection of subcutaneously transplanted MIN6 cells.

## 1. Introduction

Type 1 diabetes is a chronic autoimmune condition characterized by insulin deficiency caused by the destruction of insulin-producing pancreatic β-cells [1]. Hence, people with type 1 diabetes depend on exogenous insulin to survive and control their blood glucose. However, insulin therapy has several drawbacks, including hypoglycemia, weight gain, injection site reactions, inconvenience, and cost [2]. In contrast, pancreas and islet transplantation can provide stable and consistent glycemic control and eliminate the need for insulin injections, which can significantly improve quality of life [3]. Islet transplantation offers several advantages over pancreas transplantation. In particular, it is a less invasive procedure and lower surgical risk [3]. While most recipients achieve insulin independence shortly after transplantation [4,5], sustained insulin independence is uncommon [5,6,7]. Traditionally, isolated islets are infused into the liver through the portal vein [4,5,6,7]. However, there are several drawbacks at this site, such as immediate blood-mediated inflammatory reactions (IBMIRs) and low oxygen tension, which can lead to significant loss of transplanted islets and impair graft function [8,9,10]. Alternative implantation sites, including subcutaneous tissue, muscle, the peritoneal cavity, the omentum, the gastric submucosa, bone marrow, and the anterior chamber of the eye, are being explored to overcome the above limitations and improve transplantation outcomes and long-term graft survival [9,10,11]. Islet transplantation at the subcutaneous site offers several advantages, including a minimally invasive procedure; easy access for islet implantation, monitoring, and retrieval; and potential for bioengineered scaffolds [9,10,11]. However, without modification, it has poor results, possibly due to the limited vascularization and hypoxia at this site [9,10,11,12,13]. 

Biomaterials play crucial roles in the field of tissue engineering and regenerative medicine by providing scaffolds, matrices, and delivery systems that support the growth, differentiation, and function of cells and tissues [14]. Thermosensitive hydrogels are injectable at lower temperatures and undergo in situ gelation upon exposure to physiological temperatures in the body. This property enables minimally invasive delivery of encapsulated islets into the subcutaneous tissue via syringe injection, avoiding the need for invasive surgical procedures [14]. Incorporation of pro-angiogenic factors or cell adhesion peptides into a hydrogel matrix stimulates the formation of new blood vessels and capillary networks, improving oxygen and nutrient supply to transplanted islets and enhancing their survival and function [15]. While primary islets isolated from animals or human donors remain the gold standard for studying beta-cell biology, their availability can be limited, and their behavior can vary between preparations. MIN6 cells are a mouse insulinoma β-cell line that retains the ability to produce and secrete insulin, similar to primary beta cells [16]. Because of their ease of use, reproducibility, and versatility, they are commonly used in islet research, including studying biomaterial scaffolds for supporting transplanted cells. In previous studies, Matrigel, HyStem-C, and poly(ethylene glycol) methyl ether (mPEG)-poly(Ala) hydrogels were used for the subcutaneous transplantation of MIN6 cells in mice [17,18,19,20,21,22]. In immunocompromised nude mice, MIN6 cells proliferated and lowered blood glucose [19,20,21,22,23]. In contrast, MIN6 cells were rejected in immunocompetent C57BL/6, BALB/c, Black Swiss, Black Webster, C3H, and NOD mice [18,19].

In vivo imaging techniques allow us to monitor the fates and behaviors of transplanted islets within a recipient’s body over time, including tracking their locations, survival, engraftment, and functions [24,25]. Using noninvasive magnetic resonance imaging (MRI), we have detected and performed long-term monitoring of mouse islet iso- [26,27] and allo-grafts [27,28] as well as porcine neonatal pancreatic cell clusters [29] transplanted under mouse kidney capsules. Recently, we subcutaneously transplanted MIN6 cells incubated with chitosan-coated superparamagnetic iron oxide and/or transfected with luciferase into nude mice and successfully tracked the grafts using MR and bioluminescence imaging for up to 64 days [22]. Notably, these images correlated well with the graft histology [23]. In this study, we further applied bioluminescence imaging to investigate the rejection of luciferase-transfected MIN6 cells in subcutaneous tissues of C3H mice and correlated the image results with the graft histology. Our results indicated that bioluminescence imaging could precisely detect the immune rejection of grafted MIN6 cells in the C3H mice.

## 2. Materials and Methods

### 2.1. Culturing MIN6 Cells 

MIN6 cells were obtained from Professor Susumu Seino at Kobe University, Kobe, Japan. A total of 5.7 × 10^6^ MIN6 cells (passages 46–48) were cultured in 24 mL of Dulbecco’s modified Eagle’s medium (DMEM, Gibco, Grand Island, NY, USA) supplemented with 15% heat-inactivated fetal bovine serum (FBS, Gibco, Grand Island, NY, USA) and 1% penicillin–streptomycin. The MIN6 cells were then incubated at 37 °C with 5% CO_2_. The medium was replaced every 3 days, and cells were passaged every week [21,22].

### 2.2. Subcutaneous Transplantation of Matrigel-Embedded MIN6 Cells in Nude and C3H Mice

The animal experiment protocol was approved by the Institutional Animal Care and Use Committee (IACUC, No. 2021121406) of Chang Gung Memorial Hospital, Taiwan. Nude and C3H mice aged 8–12 weeks were purchased from the National Laboratory Animal Center, Taiwan, and used as recipients of transplantation. We transfected MIN6 cells with luciferase using lentiviral delivery and selection by puromycin. A total of 5 × 10^6^ luciferase-transfected MIN6 cells in 100 μL of a growth factor-reduced Matrigel solution (Corning, Corning, NY, USA) and 20 µL of an RPMI medium were slowly injected into the left flank of each mouse after the mice were anesthetized with isoflurane [21,22]. The recipients’ body weights were measured by a scale, and blood glucose was checked by tale snip using a glucose meter (Accu-Chek Guide, Roche, Mannheim, Germany) weekly post-transplantation. Hypoglycemia was defined as a blood glucose concentration < 60 mg/dL [30].

### 2.3. In Vivo Imaging of Subcutaneous Matrigel-Embedded MIN6 Cells in Nude and C3H Mice

After transplantation, serial bioluminescence images were acquired after administering luciferin in nude and C3H mice. Bioluminescence imaging was carried out using an in vivo imaging system (IVIS, Xenogen, PerkinElmer, Inc., Waltham, MA, USA).

### 2.4. Histological Analysis of Matrigel-Embedded MIN6 Cell Grafts in Nude and C3H Mice

The grafts of Matrigel-embedded MIN6 cells were removed from the mice at variable time points after transplantation. They were fixed in a 4% paraformaldehyde solution, embedded in paraffin, and then processed for sectioning. The graft sections were stained for β-cells with anti-insulin antibodies (Dako, Carpinteria, CA, USA, and Thermo Fisher, Cheshire, UK) [21,22], for cell proliferation with an anti-Ki67 antibody (Abcam, Cambridge, UK), for blood vessels with anti-CD31 (Thermo Fisher, Cheshire, UK) [21,25] and anti-SMA antibodies (Abcam, Cambridge, UK) [20,25], and for T cells with anti-CD4 (Abcam, Cambridge, UK) and anti-FoxP3 antibodies (eBioscience, San Diego, CA, USA). Rejection was evident from the graft histology, which showed very few insulin-positive cells with or without inflammatory cell infiltration [31]. 

### 2.5. Statistical Analysis

The data were expressed as means and standard deviations (M ± SD). Unpaired Student’s *t* tests and ANOVAs were applied to compare mean values between two groups and to conduct multiple comparisons, respectively. A *p*-value < 0.05 was considered statistically significant.

## 3. Results

### 3.1. Evolution of Recipients’ Blood Glucose and Body Weight Measurements after Subcutaneous Transplantation of Matrigel-Embedded MIN6 Cells in Nude and C3H Mice

**Figure 1:**
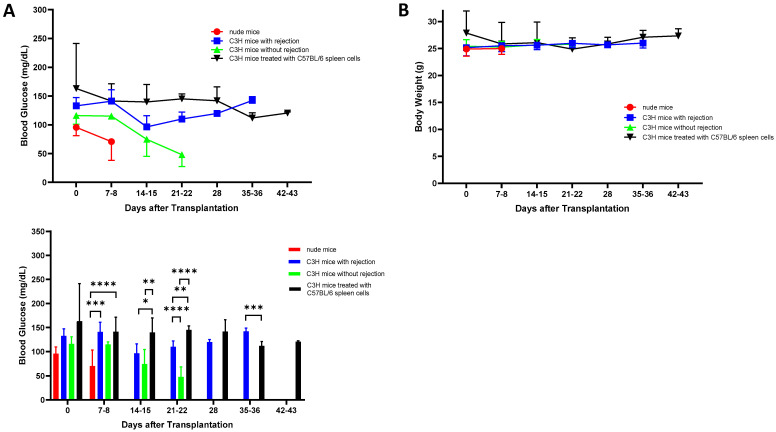
Evolution of recipients’ blood glucose (**A**) and body weight (**B**) measurements after subcutaneous implantation of 5 × 10^6^ Matrigel-embedded MIN6 cells in each nude mouse (n = 8) and C3H mouse with (n = 14) or without (n = 9) presensitization with C57BL/6 spleen cells. Rejection was evident from the graft histology. Nude mice and C3H mice without rejection were euthanized due to severe hypoglycemia by days 19 and 28, respectively. Statistics comparing the blood glucose measurements among the groups are shown in the lower panel. * *p* < 0.05, ** *p* < 0.01, *** *p* < 0.001, **** *p* < 0.0001.

After subcutaneous transplantation of Matrigel-embedded MIN6 cells, the recipients’ blood glucose declined gradually toward hypoglycemia (blood glucose < 60 mg/dL) in eight of eight (100%) nude mice and five of nine (56%) C3H mice (*p* < 0.05), with their first hypoglycemic episodes occurring at 11.6 ± 3.9 days and 14.5 ± 0.6 days, respectively (*p* = 0.1815). Hypoglycemia only occurred in C3H mice without rejection of MIN6 cells. Interestingly, after being presensitized with C57BL/6 spleen cells, none of the 14 (0%) C3H mice had hypoglycemia (*p* < 0.01 vs. C3H mice without presensitization). On days 7–8, the nude mice had lower blood glucose than the C3H mice with rejection and the C3H mice after presensitization (*p* < 0.001). Thereafter, the blood glucose levels of the C3H mice without rejection gradually decreased, and they were lower than those of the C3H mice with rejection and the C3H mice after presensitization on days 21–22 (*p* < 0.0001) (Figure 1A). In contrast, the body weight changes were not significant in the nude and C3H mice (Figure 1B).

### 3.2. In Vivo Bioluminescence Imaging of Matrigel-Embedded MIN6 Cells Subcutaneously Transplanted in Nude and C3H Mice

**Figure 2:**
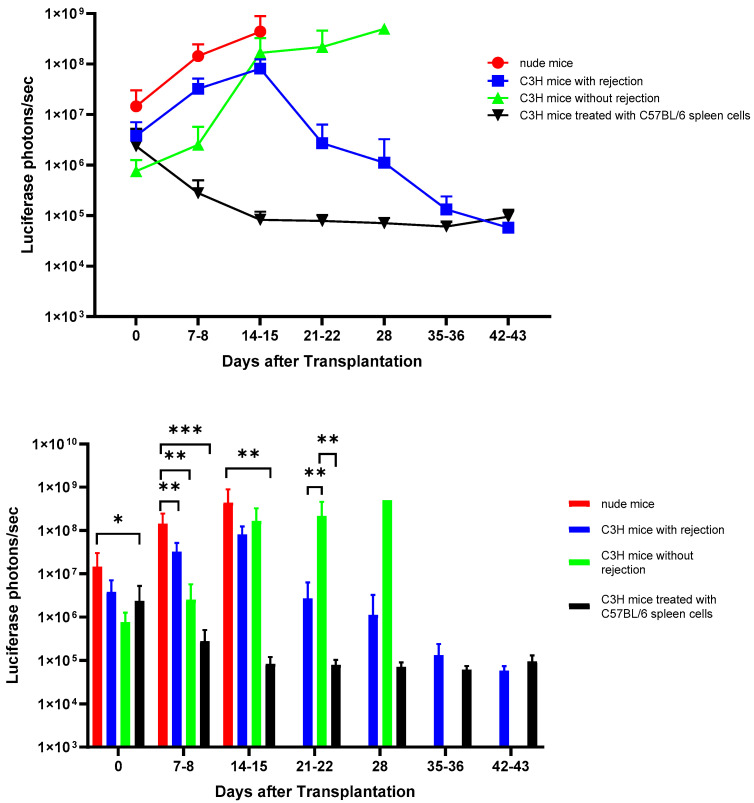
Evolution of the signal intensities (in the regions of interest (ROIs)) of the bioluminescence images of MIN6 cells after subcutaneous transplantation in the nude and C3H mice (upper panel). A total of 5 × 10^6^ luciferase-transfected MIN6 cells embedded in Matrigel were subcutaneously implanted into the left flank of each nude mouse (n = 8) and C3H mouse with (n = 14) or without (n = 9) presensitization with C57BL/6 spleen cells. The recipients were imaged by an in vivo imaging system (IVIS). Rejection was evident from the graft histology. Statistics comparing the signal intensities among the groups are shown in the lower panel. * *p* < 0.05, ** *p* < 0.01, *** *p* < 0.0001.

**Figure 3:**
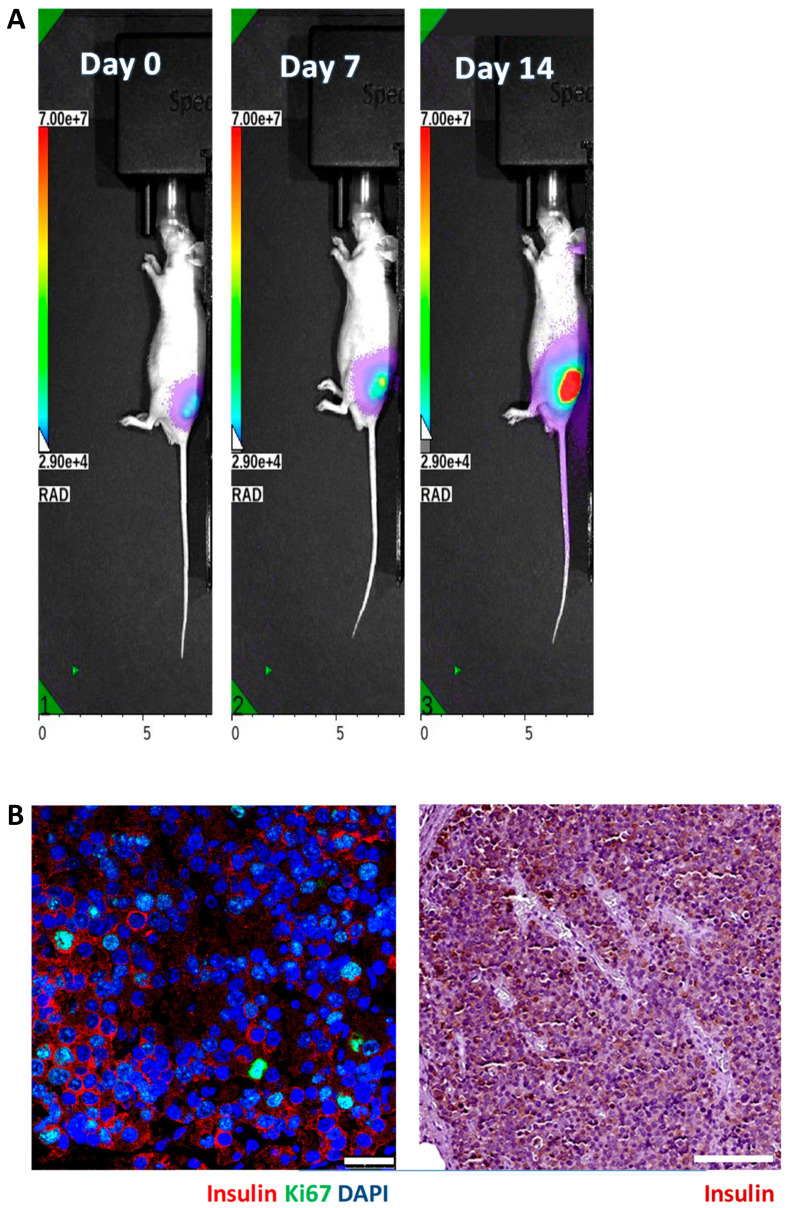
In vivo bioluminescence images (**A**) and graft histology (**B**) of MIN6 cells after subcutaneous transplantation in a representative nude mouse. A total of 5 × 10^6^ luciferase-transfected MIN6 cells embedded in Matrigel were subcutaneously implanted into the left flank of a nude mouse. (**A**) The recipient was imaged by an in vivo imaging system (IVIS). (**B**) Immunofluorescence staining of the MIN6 cell graft on day 16 for insulin (red) and ki67 (green) (left panel, scale bar: 25 µm) as well as immunoperoxidase staining for insulin (brown) (right panel, scale bar: 100 µm).

**Figure 4:**
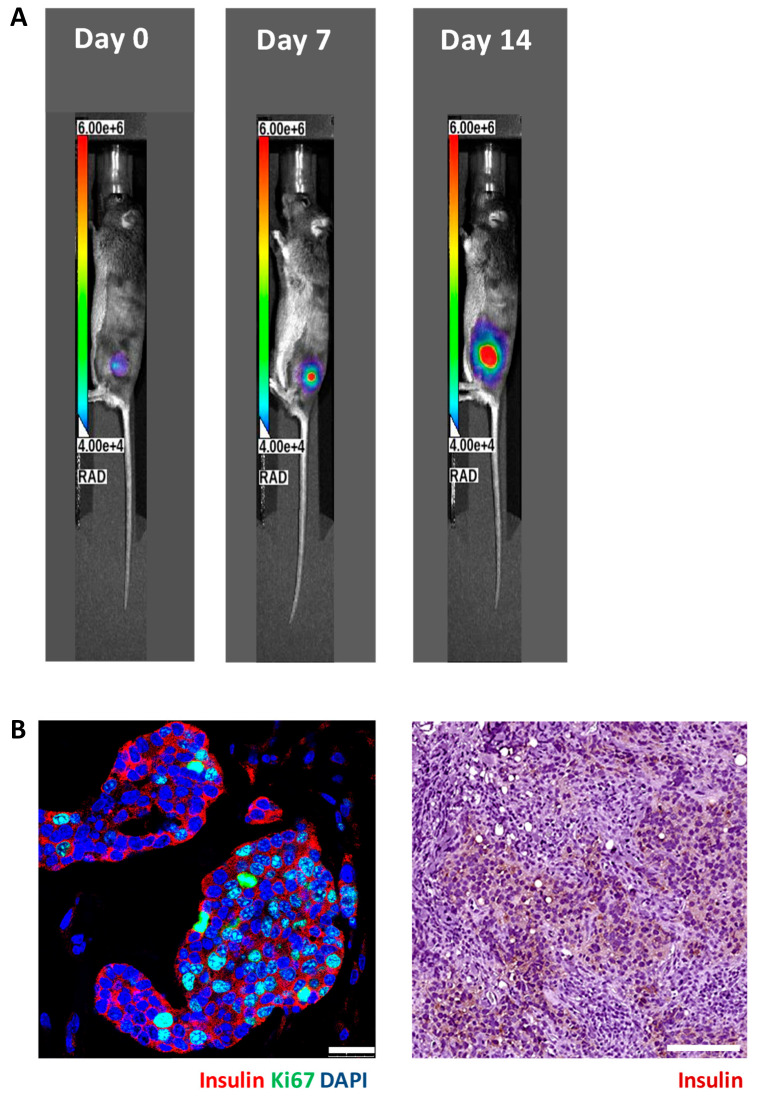
In vivo bioluminescence images (**A**) and graft histology (**B**) of MIN6 cells after subcutaneous transplantation in a representative C3H mouse without rejection. A total of 5 × 10^6^ luciferase-transfected MIN6 cells embedded in Matrigel were subcutaneously implanted into the left flank of a C3H mouse. (**A**) The recipient was imaged by an in vivo imaging system (IVIS). (**B**) Immunofluorescence staining of the MIN6 cell graft on day 14 for insulin (red) and ki67 (green) (left panel, scale bar: 25 µm) as well as immunoperoxidase staining for insulin (brown) (right panel, scale bar: 100 µm).

**Figure 5:**
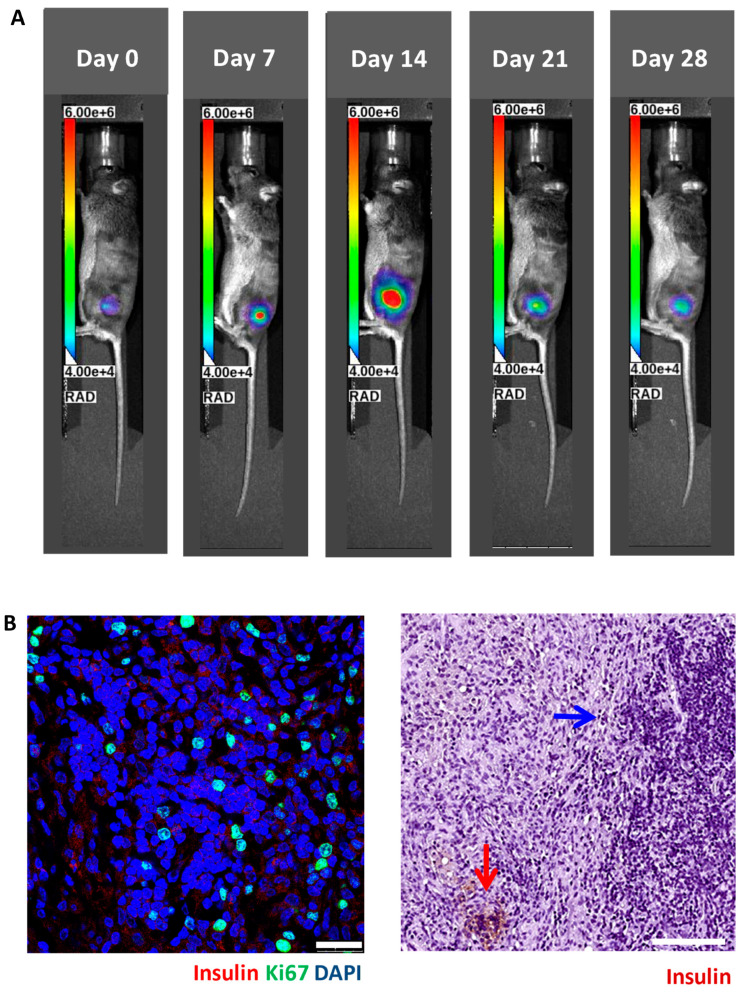
In vivo bioluminescence images (**A**) and graft histology (**B**) of MIN6 cells after subcutaneous transplantation in a representative C3H mouse with rejection. A total of 5 × 10^6^ luciferase-transfected MIN6 cells embedded in Matrigel were subcutaneously implanted into the left flank of a C3H mouse. Rejection was evident from the graft histology. (**A**) Recipients were imaged by an in vivo imaging system (IVIS). (**B**) Immunofluorescence staining of the MIN6 cell graft on day 28 for insulin (red) and ki67 (green) (left panel, scale bar: 25 µm) as well as immunoperoxidase staining for insulin (brown, indicated by a red arrow) (right panel, scale bar: 100 µm). The blue arrow indicates mononuclear cells.

**Figure 6:**
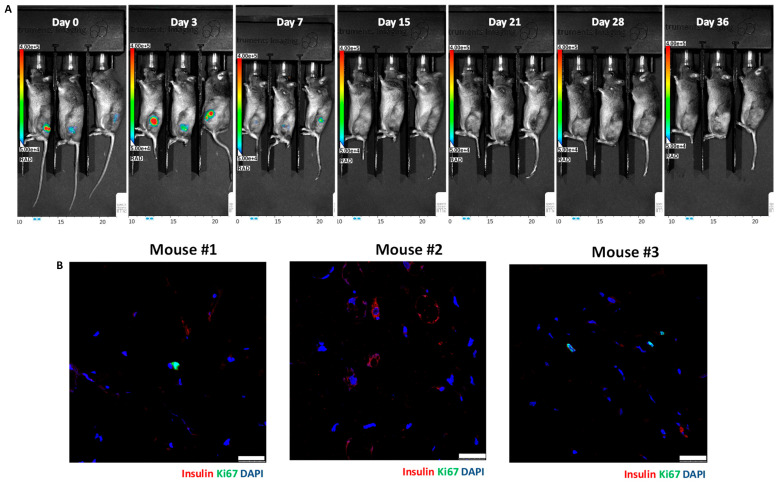
In vivo bioluminescence images (**A**) and graft histology (**B**) of MIN6 cells after subcutaneous transplantation in three representative C3H mice presensitized with C57BL/6 spleen cells. A total of 5 × 10^6^ luciferase-transfected MIN6 cells embedded in Matrigel were subcutaneously implanted into the left flank of each C3H mouse. The C3H mice were intravenously injected with C57BL/6 spleen cells just prior to transplantation. (**A**) Recipients were imaged by an in vivo imaging system (IVIS). (**B**) Immunofluorescence staining of the MIN6 cell grafts on day 41 for insulin (red) and ki67 (green). Scale bar: 25 µm.

We implanted 5 × 10^6^ luciferase-transfected MIN6 cells embedded in Matrigel into the subcutaneous space of each nude (n = 8) and C3H (n = 9) mouse. Then, serial bioluminescence images were acquired weekly. As shown in Figure 2, we observed persistent bioluminescence signals in eight of eight (100%) nude mice (Figure 3A) and five of nine (56%) C3H mice (*p* < 0.05) (Figure 4A). In the C3H mice with rejection, the bioluminescence signals reached a peak at 13.2 ± 3.0 days and then declined (Figure 5A). In the C3H mice presensitized with C57BL/6 spleen cells, 0 of 13 (0%) had persistent bioluminescence signals. Their bioluminescence signals peaked on days 0 (n = 10) and 7 (n = 3) (*p* < 0.0001 vs. C3H mice without presensitization) and then dropped rapidly (Figure 6A).

### 3.3. Histology of Subcutaneous Matrigel-Embedded MIN6 Cell Grafts in Nude and C3H Mice 

**Figure 7:**
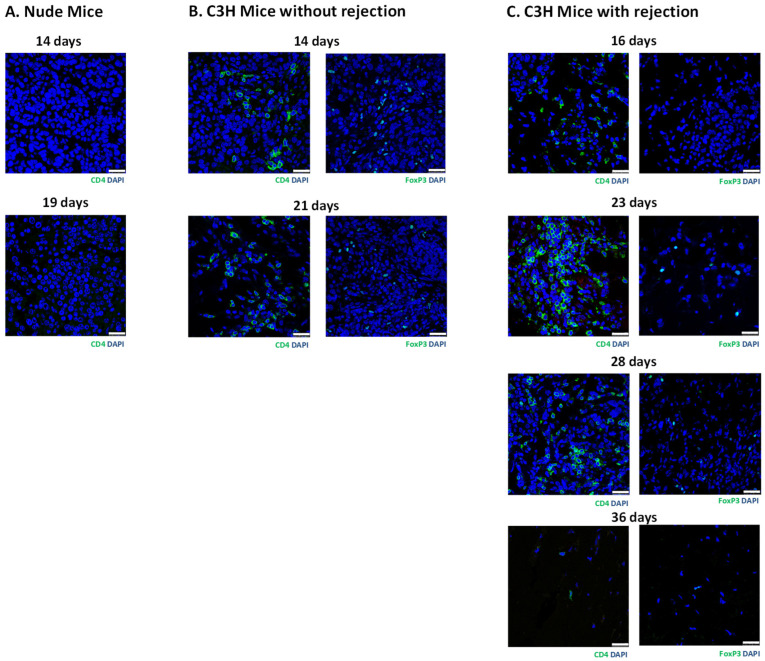
Immunofluorescence staining of MIN6 cell grafts for CD4 (green) and FoxP3 DAPI (green) in nude mice (**A**) and C3H mice without (**B**) or with (**C**) rejection. A total of 5 × 10^6^ luciferase-transfected MIN6 cells embedded in Matrigel were subcutaneously implanted into the left flank of each nude mouse and C3H mouse. Rejection was evident from the graft histology. Scale bar: 25 µm.

In grafts from the nude mice (Figure 3B) and the C3H mice without rejection (Figure 4B), we observed abundant insulin-positive and ki67-positive cells. Remarkably, these mice also exhibited hypoglycemia and persistent bioluminescence signals. In contrast, grafts from the C3H mice with rejection (Figure 5B) and the C3H mice presensitized with C57BL/6 spleen cells (Figure 6B) showed very few insulin-positive cells and lacked hypoglycemia and persistent bioluminescence signals. Notably, the 28-day MIN6 cell grafts from the C3H mice with rejections displayed marked mononuclear infiltration (right panel in Figure 5B). To characterize the T-cell subset in the MIN6 grafts, we stained the grafts with CD4 and FoxP3 antibodies. Interestingly, the 14- and 19-day grafts from the nude mice (Figure 7A) lacked CD4^+^ T cells. However, CD4^+^ T cells were observed in the 2–4-week grafts from the C3H mice without rejection (Figure 7B) and those with rejection, particularly the 23-day grafts (Figure 7C). Importantly, only the former group contained a substantial number of FoxP3^+^ T cells (Figure 7B,C). Additionally, positive staining for CD31 (an endothelial cell marker) and SMA (a vascular smooth muscle cell marker) was found in grafts from nude mice, C3H mice with and without rejection, and C3H mice with presensitization.

## 4. Discussion

Recently, we successfully utilized noninvasive MR and bioluminescence imaging to track MIN6 cells subcutaneously transplanted in immunocompromised nude mice for up to 64 days [22]. Nude mice are unable to reject grafted MIN6 cells, as they cannot generate mature T lymphocytes due to absence of a thymus. In contrast, allorejection of transplanted MIN6 cells is expected in immunocompetent mice. Using MRI, Evgenov et al. showed gradually decreasing MR signal intensity of Feridex-labeled intrahepatic human islets in Balb/c mice [31]. However, imaging studies focusing on the immune rejection of subcutaneously grafted MIN6 cells are lacking. In this study, we implanted luciferase-transfected MIN6 cells in the subcutaneous tissues of nude and C3H mice. Soon after the transplantation of the MIN6 cells, the bioluminescence signals increased in the nude and C3H mice because of the proliferation of the MIN6 cells [23]. All nude mice and about half of the C3H mice showed persistent bioluminescence signals, suggesting tolerance of the implanted MIN6 cells. In contrast, for the other half of the C3H mice, the bioluminescence signals reached a peak at 2 weeks and then declined, indicating rejection of the MIN6 cells. Moreover, the presensitization of C3H mice with C57BL/6 spleen cells hastened the loss of bioluminescence signals, indicating the acceleration of rejection, as shown in previous studies [18,19]. Importantly, the above image findings are well correlated with the graft histology. Thus, bioluminescence imaging is a useful tool to detect and monitor the rejection of subcutaneously grafted MIN6 cells. As far as we know, we are the first to utilize bioluminescence imaging to detect the allorejection of subcutaneously implanted MIN6 cells. Previously, Fowler et al. transplanted luciferase-transfected murine or human islets into the livers or under the renal capsules of immunodeficient mice and observed that bioluminescence signals were present at those sites for more than 8 weeks and were correlated with the number of transplanted islets. However, the bioluminescence signals in the kidney were approximately four times greater than those in the liver, indicating the influence of the optical absorption of the generated light and the location of the light source. Moreover, luciferase-expressing murine islets had greater in vitro and in vivo bioluminescence signals than human islets. This may have been due to species differences in the efficiency of luciferase transfection, islet purity, etc. [32]. Since a major limitation of bioluminescence imaging is that the transmission of signals can be significantly attenuated by tissue absorption and scattering, limiting the ability to detect signals from deep within the body, bioluminescence imaging can only be applied in small animal models [33].

Since MIN6 cells are insulin-secreting tumor cells [16] that proliferate and secrete insulin autonomously in vivo [19,23], all of our nude mouse recipients experienced hypoglycemia within 2 weeks after implantation of MIN6 cells embedded in Matrigel. This was faster than the time observed for MIN6 cells embedded in mPEG-poly(Ala) hydrogels [22], suggesting that different hydrogels may influence the growth and/or function of embedded cells. In contrast, hypoglycemia developed in 56% and 0% of mice without and with presensitization, respectively. These results are consistent with those of Sobel et al. [19]. Importantly, all mice with hypoglycemia also showed positive signals in the bioluminescence images and insulin-positive cells in the grafts, indicating tolerance of the MIN6 cells. In contrast, all C3H mice without hypoglycemia showed loss of signals in the bioluminescence images and insulin-positive cells in the grafts, implying MIN6 cell rejection. Accordingly, hypoglycemia is a good indicator of the tolerance of implanted MIN6 cells, although a definite diagnosis should be based on graft histology. For clinical applications, hypoglycemia should be avoided after transplantation of MIN6 cells. For this reason, Tsujimura et al. combined the herpes simplex virus thymidine kinase gene and ganciclovir to regulate the proliferation and function of MIN6 cells after transplantation in diabetic mice [34].

Previously, Kimura et al. transplanted MIN6 cells into streptozotocin-diabetic C3H mice and defined rejection as a blood glucose concentration over 300 mg/dL [18]. Sobel et al. considered the development of hypoglycemia as an indicator of tolerance of the transplanted MIN6 cells [19]. In this study, we defined the tolerance or rejection of implanted MIN6 cells by graft histology [31] because we implanted MIN6 cells into nondiabetic C3H mice and blood glucose levels may be influenced by numerous factors. To investigate the growth of subcutaneous MIN6 cells, we stained their grafts with insulin and ki67 (a proliferation marker) antibodies. The grafts from the nude mice and the C3H mice without rejection showed abundant insulin- and ki67-positive cells, indicating that the MIN6 cells were proliferating. In contrast, very few or no insulin-positive cells were observed in the grafts from the C3H mice with rejection and the C3H mice presensitized with C57BL/6 spleen cells. In addition, we also found mononuclear cell infiltration in a 28-day graft with rejection, implying the inflammatory destruction of MIN6 cells. Notably, the presence or absence of hypoglycemia and persistent bioluminescence signals correlated well with the graft histology of tolerance or rejection. In other words, a lack or minimal presence of insulin-positive cells was observed in recipients without hypoglycemia and persistent bioluminescence signals. In contrast, recipients with hypoglycemia and persistent bioluminescence signals showed a significant presence of insulin-positive cells in their grafts. Taken together, both hypoglycemia and bioluminescence images can be used to predict the tolerance or rejection of MIN6 cells.

Very few insulin-positive cells with marked mononuclear infiltration were observed in the 28-day MIN6 cell grafts from the C3H mice with rejection, which suggests inflammatory destruction of the grafts. Recognition of donor alloantigens by recipient T lymphocytes initiates immune attacks on allografts. Although CD4^+^ helper and CD8^+^ cytotoxic T cells play important roles in allorecognition, Kimura et al. found that CD4^+^ and CD8^+^ T-cell subsets were almost equal in MIN6 cell grafts with and without presensitization [18]. Since CD4^+^ (instead of CD8^+^) cells are essential for allorejection [35] and CD4^+^FoxP3^+^ regulatory T cells induce immune tolerance to alloantigens [36], we stained MIN6 cell grafts with CD4 and FoxP3 antibodies. It is reasonable that CD4^+^ T cells were found in the grafts from immunocompetent C3H mice without and with rejection but not immunocompromised nude mice. However, the former contained a substantial number of FoxP3^+^ T cells, supporting the maintenance of graft immune tolerance. In contrast, the grafts from C3H mice with rejection had very few or no FoxP3^+^ T cells, indicating no immune protection. We and others [18,19] have shown that MIN6 cells presensitized with C57BL/6 spleen cells are rejected in C3H mice. This is a good model to test strategies for preventing graft rejection, such as immunosuppressants [19], irradiation [19], and microencapsulation [37]. Moreover, bioluminescence imaging can be a useful tool for evaluating the efficacy of these interventions after transplantation.

## 5. Conclusions

Recently, we successfully utilized noninvasive MR and bioluminescence imaging to track MIN6 cells in subcutaneous tissue in nude mice for up to 64 days. Here, we further applied bioluminescence imaging to investigate the rejection of luciferase-transfected MIN6 cells in subcutaneous tissue in C3H mice and correlated the image results with the graft histology. Our results indicate that rejection of MIN6 cells occurred in C3H mice and could be enhanced by presensitization with C57BL/6 spleen cells. Moreover, the absence of hypoglycemia and a loss of bioluminescence signals after transplantation are two useful indicators for detecting the rejection of subcutaneous MIN6 cells.

## Data Availability

The original contributions presented in the study are included in the article, further inquiries can be directed to the corresponding authors.
